# Cytomegalovirus viremia associated with systemic thrombotic microangiopathy after kidney transplantation: a case report

**DOI:** 10.3389/fmed.2026.1910374

**Published:** 2026-07-28

**Authors:** Andres David Sastre-Martínez, H. A. Nati-Castillo, Carlos Fernando Acuña-Roldan, Marlon Arias-Intriago, Ronnie Calupiña-Larco, Diego A. Lucero-Guanga, Jhan S. Saavedra-Torres, Juan S. Izquierdo-Condoy

**Affiliations:** 1 Internal Medicine Department, Universidad del Valle, Cali, Colombia; 2 Grupo de Investigación en Biomateriales y Biotecnología - BEO, Facultad de Salud, Universidad Santiago de Cali, Cali, Colombia; 3 Organ and Tissue Transplant Unit, Clínica Imbanaco, Cali, Colombia; 4 One Health Research Group, Universidad de las Américas, Quito, Ecuador; 5 Departamento de Urología, Hospital Interzonal General de Agudos Evita Pueblo de Berazategui, Buenos Aires, Argentina; 6 Health Faculty, Universidad Libre, Seccional Cali, Cali, Colombia; 7 Fundación Universitaria Visión de las Américas, Grupo de investigación Biomedicina, Pereira, Colombia

**Keywords:** case report, complement activation, cytomegalovirus, endothelial injury, kidney transplantation, thrombotic microangiopathy

## Abstract

**Background:**

Post-transplant thrombotic microangiopathy (PT-TMA) is an uncommon but severe complication of kidney transplantation associated with graft dysfunction and poor outcomes. Although infections have been recognized as potential triggers, systemic thrombotic microangiopathy associated with cytomegalovirus (CMV) infection remains rarely reported. Experimental and clinical evidence suggests that CMV may promote endothelial injury and complement activation, mechanisms that could contribute to the development of microangiopathic processes in transplant recipients.

**Case presentation:**

We report the case of a 78-year-old kidney transplant recipient who developed *de novo* systemic thrombotic microangiopathy associated with CMV infection. The patient presented with thrombocytopenia, microangiopathic hemolytic anemia, and progressive graft dysfunction. Drug-induced TMA related to recent tacrolimus initiation was initially suspected; however, hematologic abnormalities persisted despite calcineurin inhibitor withdrawal. Further evaluation revealed significant CMV viremia (54,000 copies/mL), while additional viral, gastrointestinal infectious, and autoimmune evaluations did not identify a more likely alternative explanation. Intravenous ganciclovir therapy was initiated and later transitioned to oral valganciclovir. Following comprehensive management, including withdrawal of tacrolimus and targeted antiviral therapy, hemolysis resolved, platelet counts normalized, CMV viral load progressively declined until clearance, and renal graft function improved. Immunosuppression was subsequently reintroduced using a calcineurin inhibitor–free regimen with belatacept.

**Conclusion:**

CMV infection should be considered a plausible trigger of *de novo* post-transplant thrombotic microangiopathy, particularly in patients with persistent disease despite withdrawal of calcineurin inhibitors. Although post-transplant TMA is frequently multifactorial, early recognition of CMV viremia and timely comprehensive management addressing all contributing factors may be associated with favorable hematologic recovery and improvement of graft function.

## 1 Introduction

Thrombotic microangiopathy (TMA) is a severe clinical syndrome characterized by microangiopathic hemolytic anemia (MAHA), thrombocytopenia, and subsequent organ injury (1). This process results from endothelial injury and platelet activation, leading to microvascular occlusion and dysfunction of multiple organs, particularly the kidneys, central nervous system, and heart (2). TMAs are broadly classified into primary forms, including thrombotic thrombocytopenic purpura (TTP), Shiga toxin–associated hemolytic uremic syndrome (STEC-HUS), and complement-mediated hemolytic uremic syndrome (CM-HUS), and secondary forms triggered by various conditions such as drugs, pregnancy, malignant hypertension, infections, malignancies, or solid organ transplantation (3, 4).

Kidney transplantation represents a relevant clinical setting for the development of TMA. Post-transplant thrombotic microangiopathy (PT-TMA) is an uncommon but potentially devastating complication associated with graft dysfunction and an increased risk of graft loss (4, 5). PT-TMA can be further classified as recurrent TMA, typically associated with complement-mediated HUS due to complement gene abnormalities and accounting for approximately 5–10% of cases, and *de novo* TMA, which is more common (90–95%) and is usually triggered by ischemia–reperfusion injury, exposure to immunosuppressive agents (especially calcineurin inhibitors), infections, or antibody-mediated rejection (6–8).

Among the precipitating factors of PT-TMA, bacterial infections have been more frequently described as triggers of thrombotic microangiopathy in transplant recipients (9). However, viral infections may also contribute to this process through multiple mechanisms, including immune activation, endothelial dysfunction, and complement activation. In this context, viruses such as BK virus, human immunodeficiency virus, parvovirus B19, and cytomegalovirus have been implicated in cases of secondary TMA, particularly in immunocompromised patients (8, 9).

Cytomegalovirus (CMV) is one of the most common opportunistic infections in kidney transplant recipients and is associated with a wide spectrum of systemic complications, including invasive disease, graft rejection, and endothelial dysfunction (10). Although CMV infection has been described as a potential trigger of thrombotic microangiopathy in the transplant setting, most reported cases correspond to TMA limited to the renal allograft. In contrast, systemic thrombotic microangiopathy associated with CMV infection has been reported far less frequently in the literature (11, 12). Furthermore, the pathophysiological mechanisms underlying this association, particularly those involving endothelial injury and complement activation, remain incompletely understood (13, 14).

We report a case of *de novo* systemic thrombotic microangiopathy associated with cytomegalovirus infection in a kidney transplant recipient, highlighting the diagnostic approach, therapeutic decisions, and clinical outcome, as well as the potential pathophysiological implications of CMV-induced endothelial injury.

## 2 Case presentation

A 78-year-old woman with a history of hypothyroidism and treated cutaneous squamous cell carcinoma underwent a deceased-donor kidney transplantation in 2018 for end-stage renal disease secondary to autosomal dominant polycystic kidney disease. The transplant was ABO-compatible, and both donor and recipient CMV serostatus were D−/R+, respectively. Her post-transplant course had been stable for several years. Maintenance immunosuppression consisted of sirolimus, mycophenolate mofetil, and low-dose prednisone, with stable baseline graft function. She received valganciclovir prophylaxis for six months after transplantation. No previous episodes of CMV infection or disease had been documented, and she was not receiving antiviral prophylaxis at the time of presentation.

Five years after kidney transplantation, in August 2023, during routine outpatient follow-up, the patient developed lower urinary tract symptoms associated with worsening renal function. Urine culture grew extended-spectrum beta-lactamase–producing (ESBL) *Escherichia coli*, prompting hospital admission. A computed tomography urography demonstrated grade II hydronephrosis secondary to ureterovesical junction stenosis. She was treated with intravenous meropenem with clinical improvement and was scheduled for elective surgical ureteral reimplantation. In preparation for surgery and due to concerns regarding wound healing, sirolimus was discontinued and tacrolimus was initiated.

Several days later, the patient was readmitted for the planned urological procedure; however, at admission she reported fever, oral ulcers, and non-bloody diarrhea. On presentation, she was hypotensive (83/57 mmHg), tachycardic (130 beats/min), and febrile (38.5 °C), consistent with septic shock. Broad-spectrum antibiotics were initiated, and blood cultures grew ESBL-producing *E. coli*. Stool cultures and a multiplex gastrointestinal PCR panel were negative. She responded to antimicrobial therapy, although her hospital course was complicated by multiple thrombotic events, including deep vein thrombosis and pulmonary embolism, for which anticoagulation with renal-adjusted low-molecular-weight heparin (enoxaparin) was initiated.

In the subsequent weeks, during recovery from the infectious episode, the patient developed recurrent non-bloody diarrhea. Repeated stool cultures and gastrointestinal PCR panels remained negative. Concurrent laboratory evaluation revealed progressive thrombocytopenia and normocytic anemia with biochemical evidence of hemolysis, including elevated lactate dehydrogenase, undetectable haptoglobin, reticulocytosis, and the presence of schistocytes on peripheral blood smear. Renal function deteriorated in parallel. This constellation of findings was consistent with thrombotic microangiopathy. ADAMTS13 activity and baseline coagulation studies, including prothrombin time and activated partial thromboplastin time (aPTT), were within normal limits (Table 1). Fibrinogen and D-dimer levels were not obtained because of the patient’s recent thromboembolic events. Although the patient received renal-adjusted low-molecular-weight heparin (enoxaparin), the clinical course immediately following anticoagulation initiation was not suggestive of heparin-induced thrombocytopenia, as no further abrupt decline in platelet count or new thrombotic manifestations occurred.

**Table 1:** Laboratory findings during hospitalization.

Laboratory test (Unit)	Reference range	09/18/2023 (Pre-TMA)	10/23/2023 (TMA Onset)	11/14/2023 (Post-treatment)
*Hematologic parameters*				
Platelets (10^3^/μL)	150–500	214	73	128
Hemoglobin (g/dL)	11.2–15.7	12.4	9.8	12.6
Lactate dehydrogenase (U/L)	120–246	–	466	353
Haptoglobin (mg/dL)	30–200	–	<10	–
Reticulocytes (%)	0.5–1.7	–	4.37	1.72
Peripheral blood smear	–	–	Schistocytes present, Anisocytosis, Macrocytes, Microcytes, Echinocytes	–
ADAMTS-13 activity (%)	>20%	–	90.5	–
Direct coombs test	Negative	–	Negative	–
Prothrombin time (s)	9.0–11.5	–	11.0	–
Activated partial thromboplastin time (aPTT) (s)	25.3–33.8	–	25.6	
*Renal function*				
Creatinine (mg/dL)	0.52–1.04	2	1.57	0.97
*Infectious markers*				
CMV Viral Load (copies/mL)	Not Detected	–	54,000	1,405 (11/02/2023), Undetected (11/09/2023)
*Other relevant markers*				
Leukocytes (10^3^/μL)	4–10	9.45	4.11	6.51
Antinuclear antibodies	Negative	–	Negative	–
Tacrolimus trough level (ng/mL)	6–10		9.58	

Given the recent initiation of tacrolimus and a trough level of 9.58 ng/mL at the time of TMA diagnosis, drug-induced TMA was initially suspected, and the calcineurin inhibitor was discontinued. However, hematologic abnormalities and renal dysfunction persisted despite withdrawal of tacrolimus, prompting further evaluation for secondary causes. Blood pressure remained within the patient’s usual range throughout the diagnostic evaluation, making malignant hypertension unlikely. Quantitative real-time polymerase chain reaction for cytomegalovirus performed on plasma revealed a viral load of 54,000 copies/mL. The specific commercial PCR platform was not available from the retrospective medical record. Laboratory testing for other transplant-related viral infections, including BK virus, hepatitis C virus, and HIV, was negative. Based on the presence of systemic clinical and laboratory features consistent with thrombotic microangiopathy, together with significant cytomegalovirus viremia and a comprehensive evaluation that did not identify a more likely alternative etiology, CMV-associated *de novo* post-transplant thrombotic microangiopathy was considered the most plausible diagnosis.

**Figure 1:**
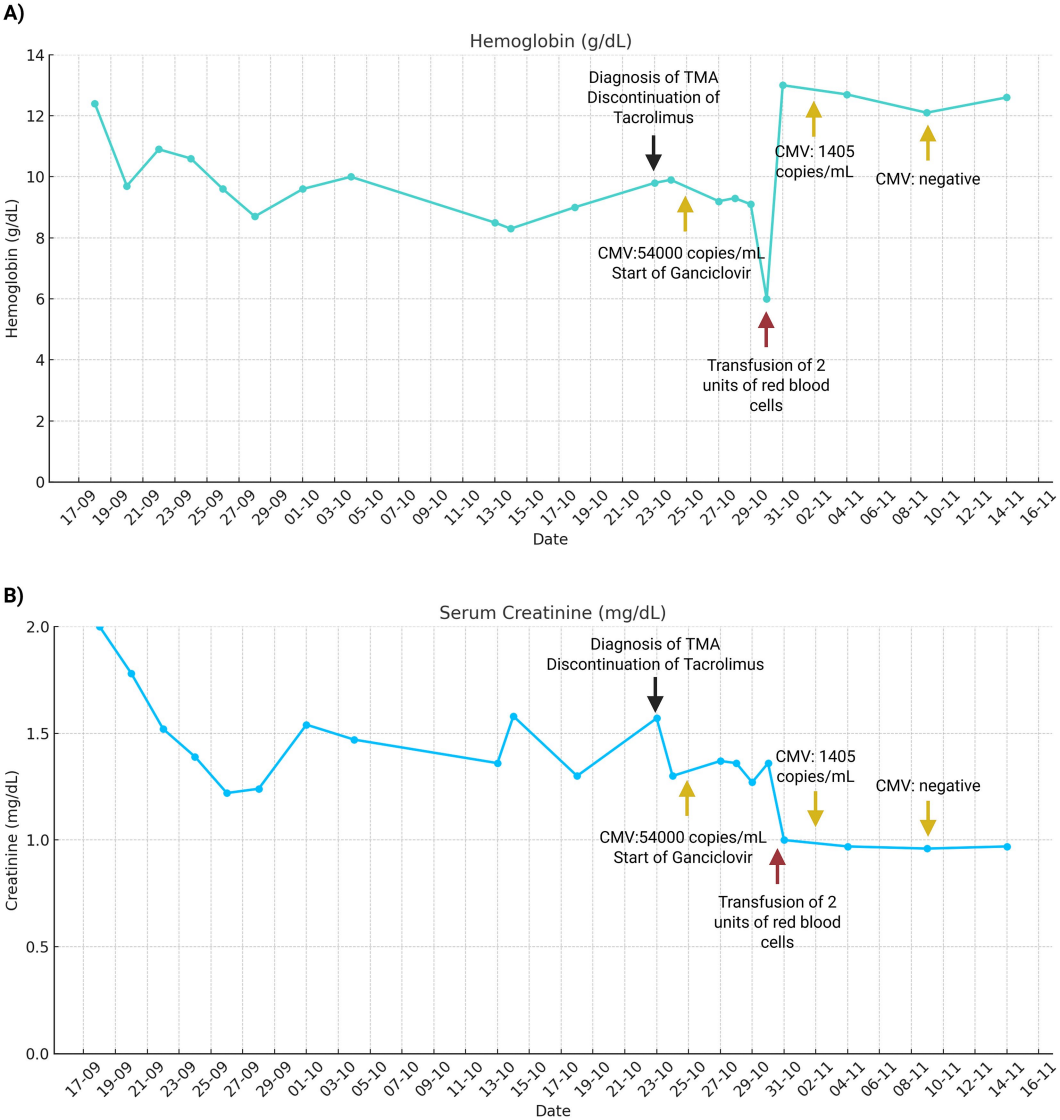
Temporal evolution of hemoglobin levels **(A)** and serum creatinine **(B)** in relation to cytomegalovirus viral load and therapeutic interventions.

Antiviral therapy with intravenous ganciclovir at a dose of 2.5 mg/kg every 24 h, adjusted for renal function, was initiated. Shortly thereafter, the patient required transfusion of two units of packed red blood cells. Over the following days, hemoglobin levels stabilized, platelet counts progressively recovered, schistocytes disappeared from peripheral blood smear, and renal function improved. Serial monitoring demonstrated a marked decline in CMV viral load to 1,405 copies/mL, followed by complete viral clearance (Figure 1). Antiviral therapy was subsequently switched to oral valganciclovir, which was continued as secondary prophylaxis for three months.

After clinical stabilization and resolution of gastrointestinal symptoms, maintenance immunosuppression was reintroduced using a calcineurin inhibitor–free regimen. Belatacept was initiated intravenously following the standard induction schedule (10 mg/kg on days 1, 5, 15, and 30), in combination with mycophenolate mofetil and prednisone. Given the known association of calcineurin inhibitors and mTOR inhibitors with thrombotic microangiopathy, these agents were intentionally avoided.

**Figure 2:**
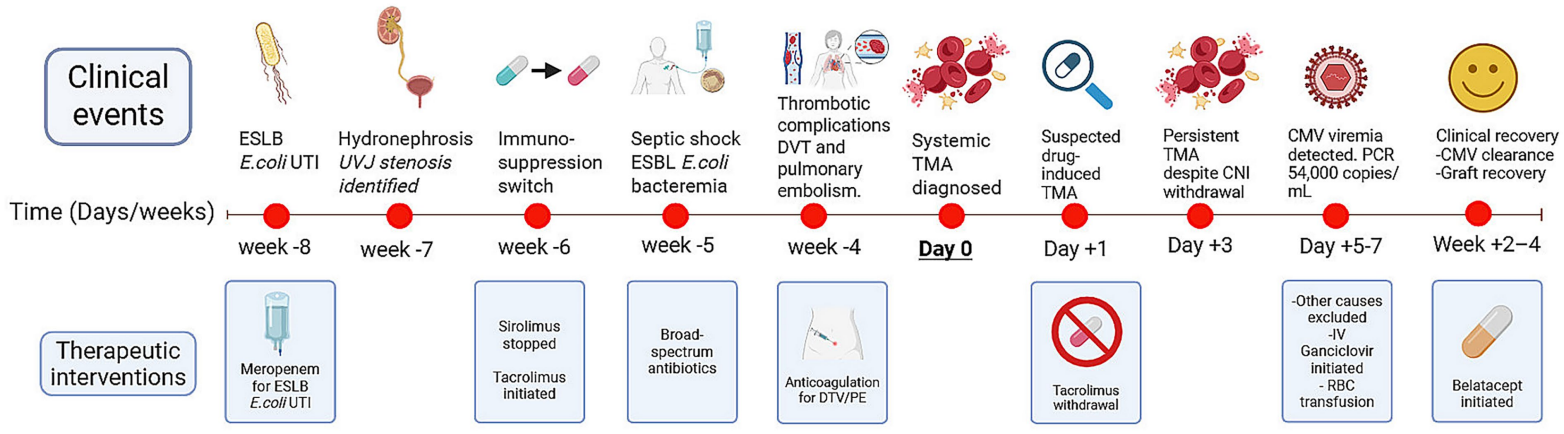
Clinical timeline summarizing major infectious events, immunosuppressive modifications, onset of systemic thrombotic microangiopathy, diagnostic evaluation, therapeutic interventions, and clinical recovery. Abbreviations: CMV, cytomegalovirus; CNI, calcineurin inhibitor; DVT, deep vein thrombosis; ESBL, extended-spectrum *β*-lactamase; PCR, polymerase chain reaction; TMA, thrombotic microangiopathy; UTI, urinary tract infection; UVJ, ureterovesical junction. Created with BioRender.com.

The patient subsequently received the scheduled belatacept doses and was discharged home in stable condition. Follow-up imaging demonstrated spontaneous resolution of the ureterovesical junction stenosis with recovery of graft drainage, and the planned ureteral reimplantation was therefore no longer required. Graft function recovered progressively, accompanied by complete resolution of the thrombotic microangiopathy. A schematic timeline summarizing the patient’s clinical course, diagnostic evaluation, and therapeutic interventions is presented in Figure 2.

## 3 Discussion

The distinction between primary and secondary TMA is clinically relevant, as both therapeutic strategies and prognosis largely depend on accurate etiological classification (15, 16). In kidney transplant recipients, thrombotic microangiopathy encompasses a broad spectrum of etiologies, with a reported incidence ranging from 0.8 to 29.4% across different series and with a significant impact on graft function and survival (17–19). Graft loss rates of up to 40% have been reported in the years following a TMA episode. Among the most common triggers are antibody-mediated rejection and exposure to immunosuppressive agents, especially calcineurin inhibitors and mTOR inhibitors (20). Infections also represent an important precipitating factor and may be involved in up to 54% of cases in some series (18).

In a French cohort that evaluated 77 episodes of thrombotic microangiopathy in kidney transplant recipients, infections accounted for approximately 42% of cases, representing one of the most common secondary triggers. Within this group, both bacterial and viral infections were implicated, each accounting for approximately 21–22% of the episodes. However, the distribution of infectious triggers varied according to the timing of presentation. Bacterial infections were more frequently observed in early TMA, whereas viral infections predominated in unexpected TMA occurring during follow-up, where they accounted for approximately 32% of cases. Among the identified viruses, CMV was one of the most frequently implicated pathogens, representing nearly 12% of all TMA episodes in this series (9).

In larger cohort studies of post-transplant TMA, viral infections, including cytomegalovirus, have been identified as potential triggers of endothelial injury. In a recent retrospective analysis of 68 kidney transplant recipients with TMA, CMV infection was significantly more frequent among patients with TMA associated with secondary etiologies, supporting the role of viral infections as potential triggers of microangiopathic processes in this population (21).

The pathophysiology of cytomegalovirus-associated thrombotic microangiopathy is not fully understood; however, endothelial injury appears to play a central role in its development. CMV exhibits a marked tropism for endothelial cells, and the ability of the virus to replicate within these cells is considered an important factor for its persistence in the host (22). Viral replication and the associated immune response promote endothelial damage mediated by the activation of CD4 + and CD8 + T lymphocytes expressing high levels of the chemokine receptors CX3CR1 and CXCR3, whose respective ligands are fractalkine (CX3CL1) and IP-10 (CXCL10) (13, 23). This immune activation promotes a state of endothelial activation characterized by increased expression of adhesion molecules (VCAM-1 and ICAM-1) and the release of von Willebrand factor, facilitating platelet adhesion and microvascular thrombus formation (24, 25). Comparable infection-driven inflammatory pathways have been shown to induce endothelial dysfunction, cytokine release, and a prothrombotic microvascular milieu in other infectious conditions (26).

This marked endothelial tropism can also be explained by specific mechanisms of viral entry. In particular, infection of endothelial cells depends on the viral pentameric complex (gH/gL/UL128/UL130/UL131), which enables viral internalization through endocytic pathways and is essential for infection of endothelial and epithelial cells. In contrast, viral entry into fibroblasts occurs predominantly through the trimeric complex (gH/gL/gO), which mediates direct fusion of the viral envelope with the plasma membrane (22). Additional mechanisms of CMV-induced endothelial injury have also been described. Experimental studies have shown that CMV infection can induce mitochondrial iron overload in endothelial cells, leading to activation of the AIM2 inflammasome and endothelial pyroptosis, a form of inflammatory cell death that amplifies vascular injury (27).

**Figure 3:**
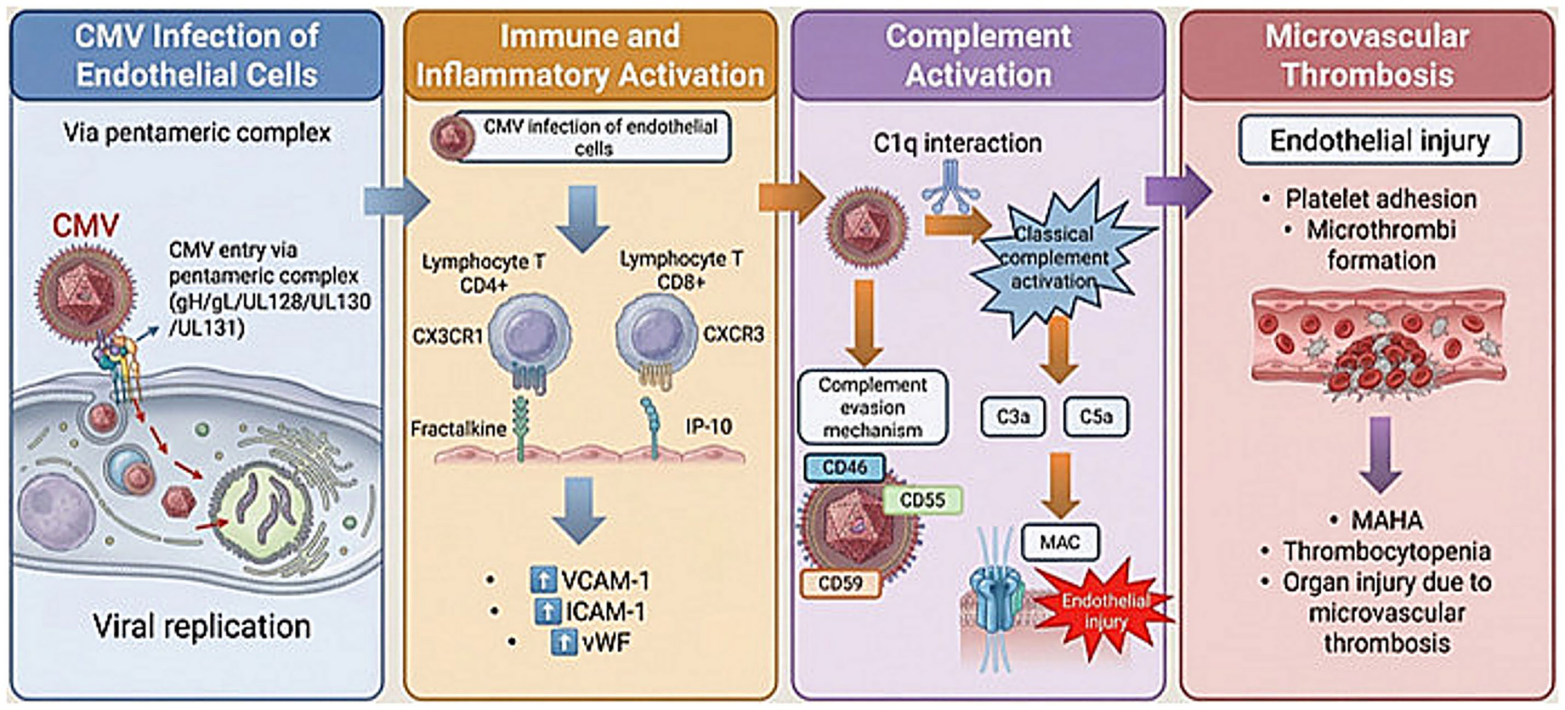
Cytomegalovirus infects endothelial cells through the pentameric entry complex (gH/gL/UL128–131), enabling viral replication within the vascular endothelium. Viral infection triggers immune and inflammatory activation, including recruitment of CD4 + and CD8 + T lymphocytes expressing CX3CR1 and CXCR3, which interact with endothelial ligands CX3CL1 (fractalkine) and CXCL10 (IP-10). This process promotes endothelial activation characterized by increased expression of adhesion molecules (VCAM-1 and ICAM-1) and release of von Willebrand factor, facilitating platelet adhesion. CMV infection can also activate the classical complement pathway through interaction with C1q, leading to generation of C3a and C5a and formation of the membrane attack complex (MAC). In parallel, CMV may evade complement-mediated neutralization by incorporating host complement regulatory proteins (CD46, CD55, CD59), allowing persistent endothelial injury. The combined effects of endothelial activation, immune signaling, and complement dysregulation promote microvascular thrombosis, resulting in systemic thrombotic microangiopathy characterized by microangiopathic hemolytic anemia, thrombocytopenia, and organ injury. Abbreviations: CMV, cytomegalovirus; CX3CR1, C-X3-C motif chemokine receptor 1; CXCR3, C-X-C motif chemokine receptor 3; CX3CL1, fractalkine; IP-10 (CXCL10), interferon gamma–induced protein 10; VCAM-1, vascular cell adhesion molecule 1; ICAM-1, intercellular adhesion molecule 1; vWF, von Willebrand factor; C1q, complement component 1q; C3a, complement component 3a; C5a, complement component 5a; CD46, membrane cofactor protein; CD55, decay-accelerating factor; CD59, protectin; MAC, membrane attack complex; MAHA, microangiopathic hemolytic anemia. Created with BioRender.com.

In addition, CMV has been shown to directly contribute to activation of the complement system. Interaction of the virus with complement components, including C1q, may primarily activate the classical pathway, leading to the generation of proinflammatory fragments such as C3a and C5a, thereby amplifying the inflammatory response and promoting endothelial injury (11). However, CMV has also developed mechanisms to evade complement-mediated neutralization. Several studies have demonstrated that the virus can incorporate host-derived complement regulatory proteins, such as CD55, CD46, and CD59, into its viral envelope. This process inhibits the formation of C3/C5 convertases and prevents assembly of the membrane attack complex (MAC). As a result, partial complement activation may occur, characterized by persistent inflammation and endothelial injury without effective viral clearance (11, 13), which may contribute to the development of thrombotic microangiopathy in CMV-infected patients. The pathophysiological mechanisms described above are summarized in Figure 3.

In the present case, primary causes of thrombotic microangiopathy were clinically evaluated and considered unlikely. Normal ADAMTS13 activity made thrombotic thrombocytopenic purpura unlikely, and no clinical or microbiological evidence supported Shiga toxin–associated hemolytic uremic syndrome. However, complement-mediated predisposition could not be completely excluded because complement levels and genetic testing were not available. Among secondary causes, calcineurin inhibitor–associated TMA was initially considered due to the recent introduction of tacrolimus. However, the persistence of hemolysis, thrombocytopenia, and worsening renal function despite withdrawal of the drug argued against calcineurin inhibitor toxicity as the sole etiological factor. Other potential secondary causes, including autoimmune disorders and alternative infectious triggers, were evaluated, although the multifactorial nature of post-transplant TMA must be acknowledged.

Cytomegalovirus (CMV) infection emerged as the most plausible trigger in the present case. CMV-associated post-transplant systemic thrombotic microangiopathy is an uncommon complication, with only a limited number of cases reported in the literature (Table 2) (11, 12, 28–32). In most reports, CMV-related TMA has been limited to the renal allograft and diagnosed histologically on kidney biopsy, often without systemic manifestations such as overt hemolytic anemia or thrombocytopenia. In contrast, systemic TMA associated with CMV infection, as observed in our patient, appears to be considerably less frequently reported in kidney transplant recipients (33). Notably, although CMV infection was identified as a trigger in nine cases within the previously mentioned French cohort of post-transplant TMA, the individual clinical characteristics of these patients were not reported, limiting detailed comparison with previously published case reports (9).

**Table 2:** Reported cases of systemic cytomegalovirus-associated thrombotic microangiopathy in kidney transplant recipients.

Author, Year	Country	Age (Years), Sex	Time after Transplant	Immunosuppression	CMV manifestation	CMV blood diagnostic tests	TMA features	Renal involvement	Other extrarenal involvement of TMA	Treatment	Dialysis required	Outcome
Jeejeebhoy et al. (1998) (29)	Canada	57, Female	6 weeks	Cyclosporine + azathioprine + prednisone	CMV pneumonitis, BAL viral culture; inclusion bodies	CMV IgM positive	MAHA, thrombocytopenia, ↑LDH, schistocytes	Acute kidney injury, no biopsy	Neurologic symptoms (headache, seizures)	CsA withdrawal, plasmapheresis (failed), IV ganciclovir	Not reported	Complete resolution
Waiser et al. (1999) (28)	Germany	28, Male	2 months	Tacrolimus + MMF + steroids	Primary CMV disease with CMV esophagitis	PCR+, pp65 antigenemia+	MAHA, ↑LDH, ↓haptoglobin, schistocytes	Acute renal failure; biopsy-proven TMA in allograft	None reported	Ganciclovir + plasmapheresis + reduction of immunosuppression	Yes	Graft failure; chronic hemodialysis
Waiser et al. (1999) (28)	Germany	33, Male	8 years	Cyclosporine + steroids	Primary CMV disease with fever and leukopenia	PCR+, pp65 antigenemia+	MAHA, thrombocytopenia, ↑LDH, schistocytes	Acute renal failure; biopsy-proven TMA	Gastrointestinal TMA (jejunal lesions)	Ganciclovir + plasmapheresis	Yes	Recovery of hemolysis but graft loss
De Keyzer et al. (2010) (30)	Belgium	46, Male	25 years	Azathioprine + prednisolone	CMV syndrome	CMV PCR: 1,600,000 copies/mL	MAHA, thrombocytopenia, ↑LDH, schistocytes	Acute graft failure; biopsy-proven TMA	None reported	Valganciclovir → IV ganciclovir + foscarnet + plasma Exchange	Yes	Persistent graft loss; chronic hemodialysis
Macesic et al. (2015) (31)	Australia	61, Male	9 months	Tacrolimus + MMF + prednisone	CMV colitis followed by CMV viremia and CMV glomerulopathy	CMV DNA PCR positive	MAHA with thrombocytopenia, elevated LDH, low haptoglobin, rare schistocytes	Biopsy-proven TMA	None reported	Plasma exchange, withdrawal of tacrolimus, CMV immunoglobulin, leflunomide, artesunate, adoptive CMV-specific T-cell therapy	Yes	Recovery of hemolysis but graft loss; chronic hemodialysis
Naidu et al. (2023) (32)	Australia	76, Female	3 years	Tacrolimus, everolimus, prednisone	CMV viremia with prior CMV colitis	CMV PCR > 300,000 copies/mL	MAHA with thrombocytopenia, elevated LDH (815 U/L), schistocytes	Acute graft failure; biopsy-proven TMA	None reported	Methylprednisolone, IVIG, plasma exchange, tacrolimus withdrawal, IV ganciclovir	Yes	Irreversible graft loss; chronic hemodialysis
Current case	Colombia	78, Female	6 years	Tacrolimus, mycophenolate mofetil, prednisone.	CMV viremia with gastrointestinal symptoms	Quantitative CMV PCR positive (54,000 copies/mL)	MAHA, thrombocytopenia, elevated LDH, undetectable haptoglobin, schistocytes; ADAMTS13 normal	Acute kidney injury without biopsy	None reported	Discontinuation of tacrolimus; IV ganciclovir followed by valganciclovir; RBC transfusion; switch to belatacept-based immunosuppression	No	Complete resolution of TMA with recovery of graft function

CMV, cytomegalovirus; TMA, thrombotic microangiopathy; MAHA, microangiopathic hemolytic anemia; LDH, lactate dehydrogenase; PCR, polymerase chain reaction; BAL, bronchoalveolar lavage; IgM, immunoglobulin M; CsA, cyclosporine A; MMF, mycophenolate mofetil; IV, intravenous; IVIG, intravenous immunoglobulin; RBC, red blood cells; ADAMTS13, a disintegrin and metalloproteinase with thrombospondin type 1 motif, member 13.

Current consensus definitions establish criteria for “proven” CMV disease in renal tissue based on viral detection by isolation, immunohistochemistry, or *in situ* hybridization in conjunction with compatible histopathological findings (10). However, no standardized diagnostic criteria exist for systemic CMV-associated TMA. In such cases, diagnosis relies on the detection of significant CMV infection in blood using validated virological assays, such as antigenemia testing (pp65 assay), viral culture, or quantitative nucleic acid amplification testing for CMV DNA (DNAemia), together with compatible clinical and laboratory findings and careful exclusion of alternative etiologies (10, 28, 34).

The management of *de novo* thrombotic microangiopathy after kidney transplantation initially focuses on identifying and correcting precipitating factors. Initial measures include reduction or discontinuation of agents potentially involved in endothelial injury, especially calcineurin inhibitors, as well as treatment of concomitant infections or episodes of acute rejection (35). In this context, early identification of the underlying trigger is crucial, as control of the precipitating factor may lead to resolution of the microangiopathic process and recovery of graft function.

In cases of TMA associated with cytomegalovirus infection, antiviral therapy represents the cornerstone of management. Nucleoside analogues that inhibit viral DNA polymerase, in particular intravenous ganciclovir or its oral prodrug valganciclovir, constitute first-line therapy in solid organ transplant recipients. Intravenous ganciclovir is typically administered at a dose of 5 mg/kg every 12 h, adjusted according to renal function, particularly in patients with severe disease or impaired gastrointestinal absorption. In clinically stable patients with mild to moderate disease, oral valganciclovir at a dose of 900 mg every 12 h may be used, with comparable efficacy in terms of viral suppression and clinical resolution (34, 36).

When CMV infection is refractory or antiviral resistance is suspected, generally defined by persistence or an increase in viral load after at least 2 weeks of adequate therapy, alternative treatments such as foscarnet or cidofovir may be considered, although their use may be limited by potential nephrotoxicity. More recently, novel antiviral agents such as maribavir, an oral inhibitor of the viral UL97 kinase, have demonstrated efficacy in the treatment of resistant or refractory CMV infections, providing a therapeutic alternative with lower hematologic and renal toxicity (34, 36, 37).

Given the central role of complement activation in many forms of post-transplant thrombotic microangiopathy, terminal complement inhibition has emerged as a relevant therapeutic strategy, particularly in severe or refractory cases. Eculizumab, a humanized monoclonal antibody targeting C5, prevents cleavage of this molecule and inhibits formation of the membrane attack complex (C5b–9), thereby reducing complement-mediated endothelial injury. Its efficacy has been well established in complement-mediated hemolytic uremic syndrome and in selected cases of post-transplant TMA associated with complement dysregulation (35). In a series including 45 patients with post-transplant TMA treated with early eculizumab therapy, rapid improvement in hematologic parameters and significant recovery of renal function were observed in a substantial proportion of patients (38). Although this cohort did not include cases of CMV-associated TMA, Java et al. reported successful treatment with eculizumab in a patient with CMV-related TMA limited to the renal allograft (11). From a pathophysiological perspective, this approach appears plausible, as CMV infection can promote complement activation and amplify endothelial injury. Therefore, complement inhibition may represent a potential therapeutic option in selected cases of CMV-associated TMA, particularly when the disease is severe, persistent, or refractory to control of the underlying infectious trigger (13, 35).

Other therapeutic strategies described in previously reported cases have included plasma exchange and intravenous immunoglobulin. However, outcomes have been heterogeneous, and graft prognosis has been unfavorable in several of the reported cases (28, 30–32). As summarized in Table 2, despite the implementation of these interventions, many patients progressed to graft loss and required chronic hemodialysis, suggesting that conventional strategies may be insufficient to reverse the microangiopathic process once it is established. In our patient, initiation of targeted antiviral therapy was temporally associated with progressive clinical remission, CMV viral clearance, and recovery of graft function. Nevertheless, the observed improvement likely reflected the combined effect of antiviral therapy, withdrawal of tacrolimus, treatment of bacterial infection, transfusion support, and subsequent optimization of immunosuppressive therapy.

Several limitations of this report should be acknowledged. Kidney biopsy and donor-specific antibody testing were not performed. In this case, kidney biopsy was deferred because of severe thrombocytopenia during the acute phase and was not subsequently pursued given the progressive recovery of graft function following treatment. Histopathological confirmation of renal TMA or tissue-invasive CMV disease, as well as definitive exclusion of antibody-mediated rejection, was therefore not possible. In addition, post-transplant TMA is frequently multifactorial, and several contributors to endothelial injury were present in our patient, including recent tacrolimus exposure, severe bacterial infection, septic shock, and CMV viremia. Although sirolimus had been discontinued approximately two weeks before the diagnosis of TMA, its potential contribution to the endothelial injury cannot be completely excluded, given the recognized association between mTOR inhibitors, endothelial dysfunction, and thrombotic complications (5). Nevertheless, the persistence of hemolysis and thrombocytopenia despite calcineurin inhibitor withdrawal, together with the marked clinical and virological response following antiviral therapy, support CMV infection as the most plausible trigger in this case. Complement studies, including complement levels and genetic testing, were not available; therefore, an underlying predisposition to complement dysregulation cannot be completely excluded.

Despite these limitations, this case underscores the importance of considering CMV infection in the differential diagnosis of *de novo* post-transplant TMA, particularly in patients with systemic manifestations and persistent disease despite withdrawal of potentially implicated immunosuppressive agents.

## 4 Conclusion

Systemic thrombotic microangiopathy associated with cytomegalovirus infection in kidney transplant recipients represents an uncommon complication whose recognition may be challenging due to the wide range of potential triggers of TMA in this clinical setting. Our case suggests that CMV infection may act as a precipitating factor for endothelial injury and systemic microangiopathic activation. Unlike several previously reported cases with unfavorable graft outcomes, our patient achieved complete resolution of hemolysis, recovery of platelet counts, and improvement of graft function after comprehensive management, including targeted antiviral therapy, withdrawal of tacrolimus, treatment of bacterial sepsis, transfusion support, and subsequent transition to a calcineurin inhibitor-free immunosuppressive regimen. These findings highlight the importance of considering CMV infection in the differential diagnosis of *de novo* post-transplant TMA and underscore that early identification and treatment of the infectious trigger may facilitate resolution of the microangiopathic process and recovery of graft function.

## Data Availability

The original contributions presented in the study are included in the article/supplementary material, further inquiries can be directed to the corresponding author.
